# 2-(2-Meth­oxy­phen­yl)-4,5-bis­(4-methyl­phen­yl)-1*H*-imidazol-3-ium 2,4,6-tri­nitro­phenolate

**DOI:** 10.1107/S2414314626005602

**Published:** 2026-06-02

**Authors:** Peter Solo, M. Arockia Doss, Michael Pillay, Tharsius Raja William Raja, Cecillia Lizhohrii, Agnes Mi, Nimchan Shimray

**Affiliations:** a Department of Chemistry, St. Joseph’s College (A), Jakhama, Nagaland, 797001, India; b Department of Environmental Studies, St. Xavier College, Jalukie, Nagaland, India; c School of Science and Humanities, St. Joseph University, Emmanuel Educity, Tindivanam-604307, India; d Department of Life and Consumer Sciences, College of Agriculture and Environmental Sciences, Florida Campus, University of South Africa, Johannesburg 1709, South Africa; e Postgraduate and Research Department of Biotechnology, Bishop Heber College (Autonomous) Tiruchirappalli Tamil Nadu - 620 017 India; Goethe-Universität Frankfurt, Germany

**Keywords:** crystal structure, hydrogen bonds, π-stacking

## Abstract

The title imidazolium picrate salt, C_24_H_23_N_2_O^+^·C_6_H_2_N_3_O_7_^−^, crystallizes in the triclinic space group *P*1. The asymmetric unit consists of one imidazolium cation and one picrate anion. The mol­ecular structure is consolidated by an intra­molecular N—H⋯O hydrogen bond within the imidazolium cation and by an inter­molecular N—H⋯O hydrogen bond between the imidazolium cation and the picrate anion. In the crystal, the ions are further associated through π–π stacking inter­actions, contributing to the supra­molecular packing arrangement. Two nitro groups of the picrate anion were modelled as disordered over two positions.

## Abstract 2

The title imidazolium picrate salt crystallizes in the triclinic space group *P*1. The asymmetric unit consists of one imidazolium cation and one picrate anion. There is an intra­molecular N—H⋯O hydrogen bond within the imidazolium cation and an inter­molecular N—H⋯O hydrogen bond between the imidazolium cation and the picrate anion. In the crystal, the ions are further associated through π–π stacking inter­actions,

## Graphical abstract

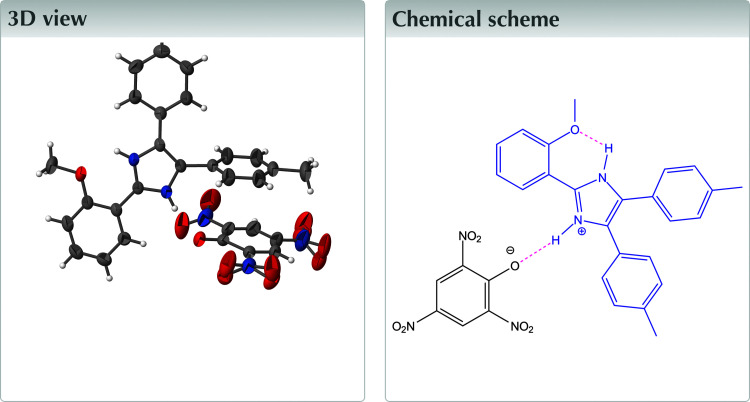

## Structure description

Imidazole and its derivatives are an important class of heterocycles in medicinal chemistry and materials science because of their diverse biological properties and their ability to participate in supra­molecular assembly (Li *et al.*, 2023 ▸). Substituted imidazolium salts are of structural inter­est since their mol­ecular conformations and crystal packing are often governed by non-covalent inter­actions such as hydrogen bonding and π–π stacking (Desiraju, 2002 ▸). Picric acid, 2,4,6-tri­nitro­phenol, is a common acidic co-former for the formation of organic salts with nitro­gen-containing bases (Bertolasi *et al.*, 2011 ▸). Related imidazolium picrate structures have also been reported previously (Du & Zhao, 2003 ▸; Dutkiewicz *et al.*, 2011 ▸; Solo *et al.*, 2025 ▸). In the present study, the crystal structure of 2-(2-meth­oxy­phen­yl)-4,5-bis­(4-methyl­phen­yl)-1*H*-imidazol-3-ium 2,4,6-tri­nitro­phenolate was determined in order to establish the mol­ecular conformation of the imidazolium cation and to examine the hydrogen-bonding and π–π stacking inter­actions responsible for the crystal packing.

**Figure 1:**
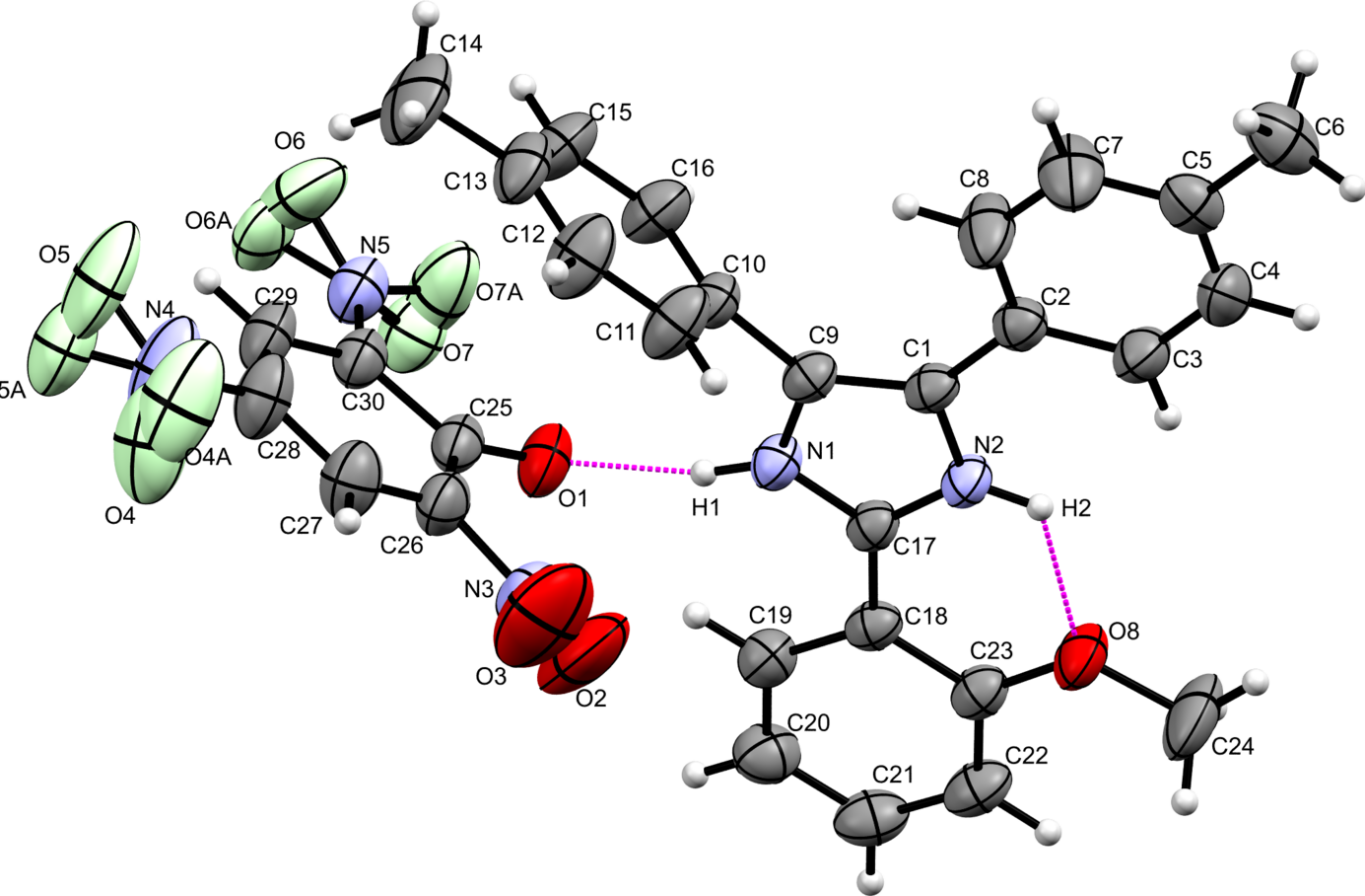
Asymmetric unit of the title imidazolium picrate salt, with displacement ellipsoids drawn at the 50% probability level. Dashed lines (magenta) indicate N—H⋯O hydrogen-bonding inter­actions. The split oxygen atoms of two nitro groups are coloured in green.

The asymmetric unit of the title salt, C_24_H_23_N_2_O^+^·C_6_H_2_N_3_O_7_^−^, contains one 2-(2-meth­oxy­phen­yl)-4,5-bis­(4-methyl­phen­yl)-1*H*-imidazol-3-ium cation and one 2,4,6-tri­nitro­phenolate anion (Fig. 1 ▸). Proton transfer from picric acid to the imidazole N atom gives the imidazolium cation and the picrate anion.

**Figure 2:**
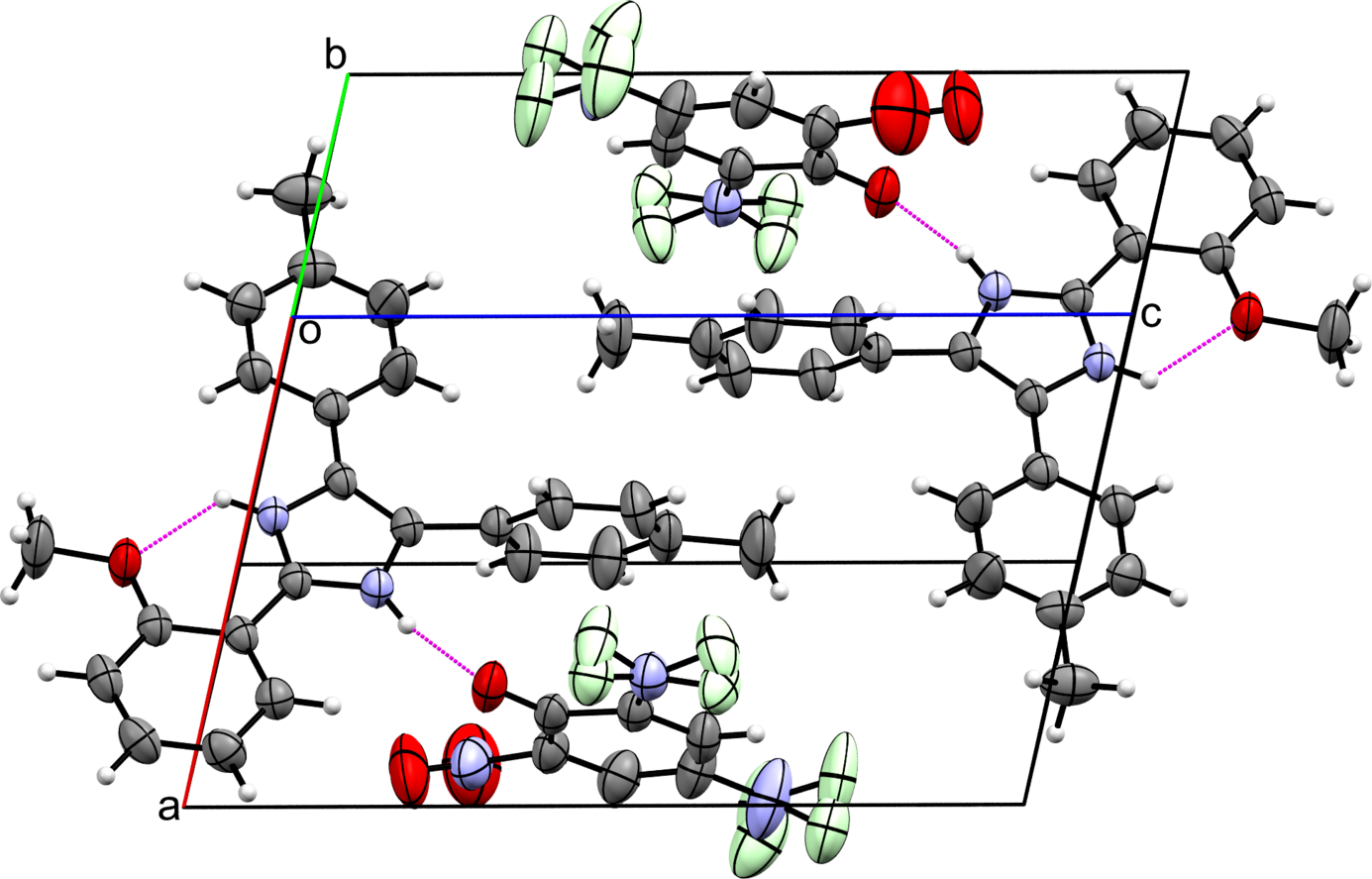
Unit-cell packing diagram of the title imidazolium picrate salt, showing the arrangement of the imidazolium cations and picrate anions in the triclinic *P*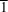 unit cell. Hydrogen-bonding inter­actions are shown as magenta dashed lines. Displacement ellipsoids are drawn at the 50% probability level.

The mol­ecular conformation is consolidated by an intra­molecular N2—H2⋯O8 hydrogen bond within the imidazolium cation, involving the imidazolium N—H group and the meth­oxy O atom. In addition, an inter­molecular N1—H1⋯O1 hydrogen bond links the imidazolium cation to the picrate anion. These N—H⋯O inter­actions help organize the cation–anion pairs and contribute to the crystal packing arrangement (Fig. 2 ▸). The hydrogen-bonding details are listed in Table 1 ▸.

**Figure 3:**
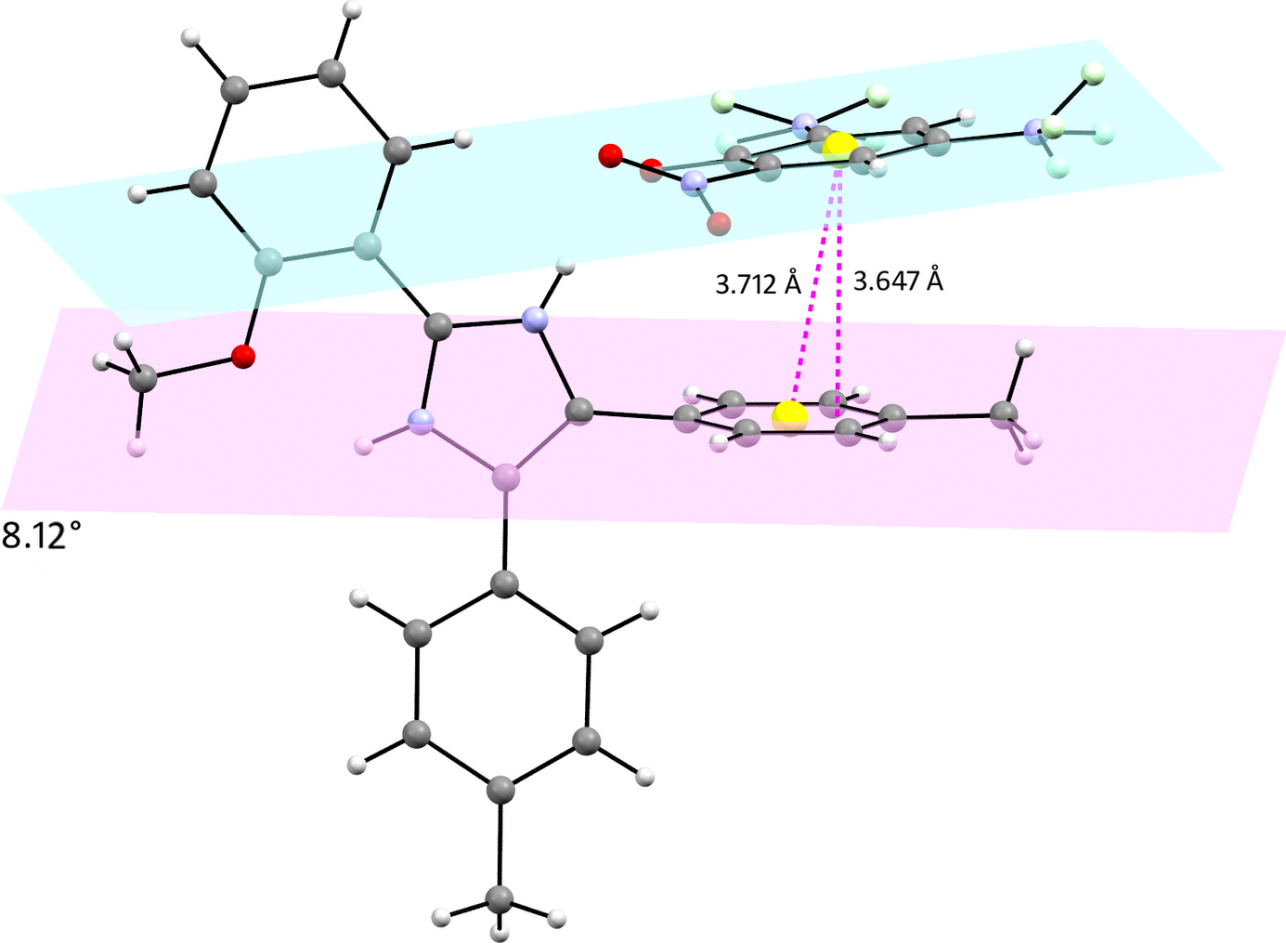
π–π stacking inter­action between the picrate ring and the *p*-tolyl ring, showing a centroid–centroid distance of 3.712 (2) Å, and a dihedral angle of 8.12 (14)°.

The packing is further consolidated by π–π stacking inter­actions between the picrate aromatic ring and an aromatic ring of the imidazolium cation (Fig. 3 ▸). The centroid–centroid separation is 3.712 (2) Å, the slippage is 0.69 Å and the dihedral angle between the inter­acting ring planes is 8.12 (14)°, indicating a nearly parallel arrangement of the aromatic rings (Janiak, 2000 ▸).

Two nitro groups of the picrate anion are disordered over two positions. The disorder was modelled using two sets of oxygen positions, with refined occupancies of 0.69 (4):0.31 (4) and 0.74 (3):0.26 (3) for the major and minor components, respectively. The disordered nitro groups were restrained to maintain chemically reasonable N—O and O⋯O distances and acceptable displacement parameters.

## Synthesis and crystallization

**Figure 4:**
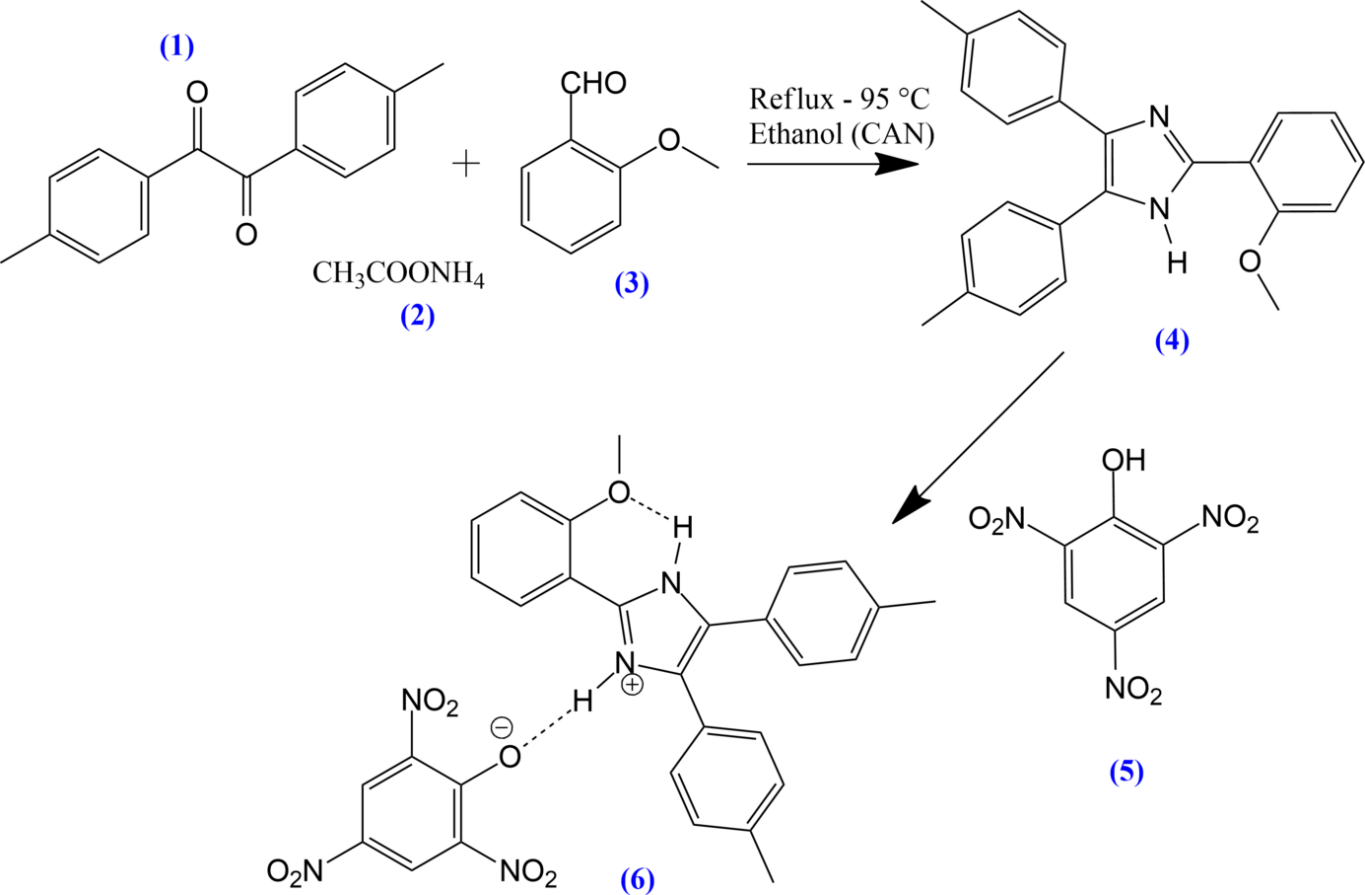
Synthesis of the title imidazolium picrate salt **(6)** from 4,4′-di­methyl­benzil **(1)**, ammonium acetate **(2)**, 2-meth­oxy­benzaldehyde **(3)** and picric acid **(5)**. The imidazole inter­mediate **(4)** was obtained in ethanol/CAN under reflux at 95°C, followed by salt formation with picric acid.

2-(2-Meth­oxy­phen­yl)-4,5-bis­(4-methyl­phen­yl)-1*H*-imidazol (**4**) was synthesized by a one-pot condensation reaction of 4,4-di­methyl­benzil (**1**) (0.953 g, 0.004 mol), 2-meth­oxy­benzaldehyde (**3**) (0.545 g, 0.004 mol), and ammonium acetate (**2**) (1.233 g, 0.016 mol) in the presence of ceric ammonium nitrate (CAN) as catalyst. Ethanol was used as the solvent and the reflux was carried out at 95°C. The progress of the reaction was monitored with TLC (hexa­ne:ethyl acetate, 1:1) and at the completion of the reaction the mixture was poured into ice-cold water. The precipitate was collected and purified with multiple recrystallization in 90% ethanol. Equimolar amounts of 2-(2-meth­oxy­phen­yl)-4,5-bis­(4-methyl­phen­yl)-1*H*-imidazol (0.071 g, 0.0002 mol) and picric acid (0.046 g, 0.0002 mol) were dissolved in 100% ethanol and heated to 120°C. The solution was kept still in a dark environment for days until yellow crystals of imidazolium picrate (**6**) appeared (Fig. 4 ▸).

## Refinement

Crystal data, data collection and structure refinement details are summarized in Table 2 ▸. Hydrogen atoms were placed in calculated positions and refined using a riding model, with methyl groups treated as rotating groups. The picrate anion showed disorder affecting two nitro groups. The O6/O7 and O4/O5 nitro oxygen atoms were modelled over two sets of positions, with refined occupancies of 0.69 (4)(major):0.31 (4)(minor) and 0.74 (3)(major):0.26 (3)(minor), respectively. The corresponding disordered atoms were assigned to PART 1 and PART 2 using linked free variables. SADI restraints were applied to maintain chemically reasonable N—O and O⋯O distances within the disordered nitro groups, while SIMU and RIGU restraints were used to restrain the anisotropic displacement parameters of the nitro-group atoms. Similar ADP restraints were also applied to the remaining nitro group to account for enlarged displacement parameters. The final weighting scheme was applied and the refinement converged with low residual electron density.

## Supplementary Material

Crystal structure: contains datablock(s) I. DOI: 10.1107/S2414314626005602/bt4200sup1.cif

Structure factors: contains datablock(s) I. DOI: 10.1107/S2414314626005602/bt4200Isup2.hkl

Supporting information file. DOI: 10.1107/S2414314626005602/bt4200Isup3.cml

CCDC reference: 2555221

Additional supporting information:  crystallographic information; 3D view; checkCIF report

## Figures and Tables

**Table 1:** Hydrogen-bond geometry (Å, °)

*D*—H⋯*A*	*D*—H	H⋯*A*	*D*⋯*A*	*D*—H⋯*A*
N1—H1⋯O1	0.86	1.82	2.661 (2)	167
N2—H2⋯O8	0.86	2.05	2.601 (2)	122

**Table 2:** Experimental details

Crystal data	
Chemical formula	C_24_H_23_N_2_O^+^·C_6_H_2_N_3_O_7_^−^
*M*_r_	583.55
Crystal system, space group	Triclinic, *P* 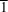
Temperature (K)	296
*a*, *b*, *c* (Å)	8.4517 (19), 12.359 (3), 13.626 (3)
α, β, γ (°)	91.961 (6), 103.983 (6), 91.860 (7)
*V* (Å^3^)	1379.1 (5)
*Z*	2
Radiation type	Mo *K*α
μ (mm^−1^)	0.10
Crystal size (mm)	0.25 × 0.18 × 0.12

Data collection	
Diffractometer	Bruker APEXII CCD
No. of measured, independent and observed [*I* > 2σ(*I*)] reflections	58614, 6492, 3785
*R*_int_	0.079
(sin θ/λ)_max_ (Å^−1^)	0.657

Refinement	
*R*[*F*^2^ > 2σ(*F*^2^)], *wR*(*F*^2^), *S*	0.051, 0.155, 1.00
No. of reflections	6492
No. of parameters	430
No. of restraints	206
H-atom treatment	H-atom parameters constrained
Δρ_max_, Δρ_min_ (e Å^−3^)	0.25, −0.16

Computer programs: *APEX2* (and *SAINT* (Bruker, 2018 ▸), *SHELXT2018/2* (Sheldrick, 2015*a* ▸), *SHELXL2025/1* (Sheldrick, 2015*b* ▸), *Mercury* (Macrae *et al.*, 2020 ▸) and *OLEX2* (Dolomanov *et al.*, 2009 ▸).

## full crystallographic data

### 2-(2-Methoxyphenyl)-4,5-bis(4-methylphenyl)-1H-imidazol-3-ium 2,4,6-trinitrophenolate Crystal data

**Table:**

C_24_H_23_N_2_O^+^·C_6_H_2_N_3_O_7_^−^	*Z* = 2
*M**_r_* = 583.55	*F*(000) = 608
Triclinic, *P*1	*D*_x_ = 1.405 Mg m^−^^3^
*a* = 8.4517 (19) Å	Mo *K*α radiation, λ = 0.71073 Å
*b* = 12.359 (3) Å	Cell parameters from 5303 reflections
*c* = 13.626 (3) Å	θ = 2.3–21.5°
α = 91.961 (6)°	µ = 0.10 mm^−^^1^
β = 103.983 (6)°	*T* = 296 K
γ = 91.860 (7)°	Block, clear yellowish orange
*V* = 1379.1 (5) Å^3^	0.25 × 0.18 × 0.12 mm

### 2-(2-Methoxyphenyl)-4,5-bis(4-methylphenyl)-1H-imidazol-3-ium 2,4,6-trinitrophenolate Data collection

**Table:**

Bruker APEXII CCD diffractometer	*R*_int_ = 0.079
φ and ω scans	θ_max_ = 27.8°, θ_min_ = 1.5°
58614 measured reflections	*h* = −11→11
6492 independent reflections	*k* = −16→16
3785 reflections with *I* > 2σ(*I*)	*l* = −17→17

### 2-(2-Methoxyphenyl)-4,5-bis(4-methylphenyl)-1H-imidazol-3-ium 2,4,6-trinitrophenolate Refinement

**Table:**

Least-squares matrix: full	Hydrogen site location: inferred from neighbouring sites
*R*[*F*^2^ > 2σ(*F*^2^)] = 0.051	H-atom parameters constrained
*wR*(*F*^2^) = 0.155	(Δ/σ)_max_ = 0.001
*S* = 1.00	Δρ_max_ = 0.25 e Å^−^^3^
6492 reflections	Δρ_min_ = −0.16 e Å^−^^3^
430 parameters	Extinction correction: SHELXL-2019/2 (Sheldrick 2015b), Fc^*^=kFc[1+0.001xFc^2^λ^3^/sin(2θ)]^-1/4^
206 restraints	Extinction coefficient: 0.0146 (18)
Primary atom site location: dual	

### 2-(2-Methoxyphenyl)-4,5-bis(4-methylphenyl)-1H-imidazol-3-ium 2,4,6-trinitrophenolate Special details

**Table:**

Geometry. All esds (except the esd in the dihedral angle between two l.s. planes) are estimated using the full covariance matrix. The cell esds are taken into account individually in the estimation of esds in distances, angles and torsion angles; correlations between esds in cell parameters are only used when they are defined by crystal symmetry. An approximate (isotropic) treatment of cell esds is used for estimating esds involving l.s. planes.
Refinement. The structure was solved with *SHELXT* 2018/2 (Sheldrick, 2015*b*) using intrinsic phasing in the triclinic space group *P*1 and refined by full-matrix least-squares on *F*^2^ using *SHELXL* 2025/1 (Sheldrick, 2015*a*) within *OLEX2*. All non-hydrogen atoms were refined anisotropically.

### 2-(2-Methoxyphenyl)-4,5-bis(4-methylphenyl)-1H-imidazol-3-ium 2,4,6-trinitrophenolate Fractional atomic coordinates and isotropic or equivalent isotropic displacement parameters (Å^2^)

**Table:**

	*x*	*y*	*z*	*U*_iso_*/*U*_eq_	Occ. (<1)
C1	0.4853 (2)	0.30529 (15)	0.09982 (13)	0.0363 (4)	
C2	0.3818 (2)	0.39655 (15)	0.06932 (14)	0.0388 (4)	
C3	0.3181 (3)	0.41538 (18)	−0.03149 (15)	0.0472 (5)	
H3	0.340672	0.368829	−0.080982	0.057*	
C4	0.2216 (3)	0.50208 (18)	−0.05992 (16)	0.0513 (6)	
H4	0.179581	0.512070	−0.128498	0.062*	
C5	0.1852 (3)	0.57407 (17)	0.00922 (17)	0.0470 (5)	
C6	0.0801 (3)	0.66833 (19)	−0.0219 (2)	0.0636 (7)	
H6A	0.080399	0.685042	−0.090154	0.095*	
H6B	0.122080	0.730101	0.022492	0.095*	
H6C	−0.029456	0.650259	−0.018000	0.095*	
C7	0.2475 (3)	0.5545 (2)	0.10897 (18)	0.0699 (8)	
H7	0.224295	0.601298	0.158085	0.084*	
C8	0.3434 (3)	0.4682 (2)	0.13924 (17)	0.0684 (8)	
H8	0.382946	0.457803	0.207963	0.082*	
C9	0.5620 (2)	0.26805 (15)	0.19136 (13)	0.0371 (4)	
C10	0.5798 (2)	0.30630 (15)	0.29722 (13)	0.0382 (4)	
C11	0.6787 (3)	0.39536 (18)	0.33662 (16)	0.0588 (6)	
H11	0.728526	0.435304	0.294933	0.071*	
C12	0.7047 (4)	0.4261 (2)	0.43808 (17)	0.0694 (7)	
H12	0.772396	0.486565	0.463553	0.083*	
C13	0.6336 (3)	0.3698 (2)	0.50166 (15)	0.0592 (6)	
C14	0.6675 (5)	0.4030 (3)	0.61299 (18)	0.0948 (11)	
H14A	0.566647	0.403341	0.633654	0.142*	
H14B	0.718963	0.474251	0.624110	0.142*	
H14C	0.738340	0.352523	0.651819	0.142*	
C15	0.5350 (3)	0.2809 (2)	0.46217 (17)	0.0644 (7)	
H15	0.485591	0.241248	0.504209	0.077*	
C16	0.5076 (3)	0.24905 (19)	0.36137 (16)	0.0546 (6)	
H16	0.439959	0.188458	0.336301	0.065*	
C17	0.6164 (2)	0.15653 (15)	0.07255 (13)	0.0347 (4)	
C18	0.6833 (2)	0.06963 (15)	0.02284 (13)	0.0364 (4)	
C19	0.7747 (3)	−0.00823 (16)	0.08002 (15)	0.0433 (5)	
H19	0.791751	−0.004604	0.150105	0.052*	
C20	0.8400 (3)	−0.09065 (18)	0.03382 (17)	0.0518 (6)	
H20	0.900649	−0.142369	0.072675	0.062*	
C21	0.8156 (3)	−0.09635 (18)	−0.06953 (17)	0.0534 (6)	
H21	0.860003	−0.152130	−0.100291	0.064*	
C22	0.7263 (3)	−0.02068 (18)	−0.12814 (16)	0.0498 (5)	
H22	0.710708	−0.025245	−0.198125	0.060*	
C23	0.6596 (2)	0.06255 (16)	−0.08264 (14)	0.0401 (5)	
C24	0.5352 (4)	0.1358 (3)	−0.24251 (17)	0.0837 (9)	
H24A	0.470770	0.195726	−0.268477	0.126*	
H24B	0.475928	0.069056	−0.268077	0.126*	
H24C	0.635683	0.139146	−0.263578	0.126*	
C25	0.8692 (3)	0.11473 (17)	0.41365 (14)	0.0450 (5)	
C26	0.9880 (3)	0.20295 (19)	0.42170 (15)	0.0517 (6)	
C27	1.0675 (3)	0.2554 (2)	0.51019 (17)	0.0627 (7)	
H27	1.141307	0.312900	0.510247	0.075*	
C28	1.0371 (3)	0.22234 (19)	0.59978 (16)	0.0628 (7)	
C29	0.9290 (3)	0.13748 (18)	0.60059 (15)	0.0551 (6)	
H29	0.910949	0.115200	0.661617	0.066*	
C30	0.8483 (3)	0.08592 (16)	0.51213 (14)	0.0442 (5)	
N1	0.6405 (2)	0.17613 (12)	0.17185 (11)	0.0380 (4)	
H1	0.696996	0.137103	0.217506	0.046*	
N2	0.52282 (19)	0.23449 (13)	0.02905 (11)	0.0380 (4)	
H2	0.489733	0.240122	−0.035337	0.046*	
N3	1.0314 (3)	0.2402 (2)	0.33090 (17)	0.0732 (6)	
N4	1.1240 (4)	0.2754 (2)	0.69380 (17)	0.1012 (10)	
N5	0.7348 (3)	−0.00302 (16)	0.51988 (14)	0.0564 (5)	
O1	0.7905 (2)	0.06989 (12)	0.33241 (10)	0.0566 (4)	
O2	1.0161 (3)	0.1779 (2)	0.25877 (16)	0.1128 (9)	
O3	1.0822 (4)	0.3330 (2)	0.33096 (19)	0.1259 (10)	
O4	1.2391 (12)	0.3402 (7)	0.6942 (9)	0.108 (2)	0.74 (3)
O5	1.070 (2)	0.2550 (9)	0.7706 (4)	0.120 (4)	0.74 (3)
O6	0.681 (2)	−0.0065 (12)	0.5949 (9)	0.095 (3)	0.69 (4)
O7	0.7000 (15)	−0.0726 (5)	0.4513 (5)	0.0689 (17)	0.69 (4)
O4A	1.189 (6)	0.366 (2)	0.687 (2)	0.133 (12)	0.26 (3)
O5A	1.157 (3)	0.2208 (14)	0.7717 (10)	0.087 (5)	0.26 (3)
O6A	0.743 (3)	−0.0316 (16)	0.6066 (8)	0.066 (4)	0.31 (4)
O7A	0.640 (5)	−0.045 (3)	0.4473 (8)	0.096 (8)	0.31 (4)
O8	0.5696 (2)	0.14085 (12)	−0.13492 (10)	0.0539 (4)	

### 2-(2-Methoxyphenyl)-4,5-bis(4-methylphenyl)-1H-imidazol-3-ium 2,4,6-trinitrophenolate Atomic displacement parameters (Å^2^)

**Table:**

	*U*^11^	*U*^22^	*U*^33^	*U*^12^	*U*^13^	*U*^23^
C1	0.0404 (11)	0.0385 (10)	0.0310 (9)	−0.0005 (8)	0.0114 (8)	−0.0056 (8)
C2	0.0404 (11)	0.0401 (11)	0.0361 (10)	0.0009 (9)	0.0100 (8)	−0.0016 (8)
C3	0.0547 (13)	0.0531 (13)	0.0370 (10)	0.0082 (11)	0.0162 (10)	0.0024 (9)
C4	0.0535 (14)	0.0588 (14)	0.0437 (11)	0.0080 (11)	0.0134 (10)	0.0161 (10)
C5	0.0401 (12)	0.0397 (11)	0.0611 (13)	−0.0011 (9)	0.0115 (10)	0.0080 (10)
C6	0.0535 (15)	0.0460 (13)	0.0926 (19)	0.0078 (11)	0.0175 (13)	0.0194 (13)
C7	0.0879 (19)	0.0646 (16)	0.0528 (14)	0.0339 (15)	0.0065 (13)	−0.0134 (12)
C8	0.0897 (19)	0.0739 (17)	0.0369 (11)	0.0383 (15)	0.0030 (12)	−0.0082 (11)
C9	0.0424 (11)	0.0370 (10)	0.0325 (9)	0.0002 (8)	0.0111 (8)	−0.0032 (8)
C10	0.0475 (12)	0.0381 (10)	0.0293 (9)	0.0041 (9)	0.0100 (8)	−0.0004 (8)
C11	0.0873 (18)	0.0495 (13)	0.0396 (11)	−0.0147 (12)	0.0188 (11)	−0.0049 (10)
C12	0.102 (2)	0.0575 (15)	0.0433 (13)	−0.0169 (14)	0.0124 (13)	−0.0134 (11)
C13	0.0885 (18)	0.0581 (14)	0.0312 (10)	0.0170 (13)	0.0135 (11)	−0.0031 (10)
C14	0.144 (3)	0.102 (2)	0.0364 (13)	0.022 (2)	0.0182 (16)	−0.0132 (14)
C15	0.093 (2)	0.0669 (16)	0.0419 (12)	0.0014 (14)	0.0328 (13)	0.0026 (11)
C16	0.0678 (15)	0.0568 (14)	0.0425 (11)	−0.0085 (12)	0.0222 (11)	−0.0029 (10)
C17	0.0370 (11)	0.0362 (10)	0.0306 (9)	−0.0013 (8)	0.0081 (8)	−0.0023 (7)
C18	0.0388 (11)	0.0368 (10)	0.0346 (9)	−0.0024 (8)	0.0124 (8)	−0.0044 (8)
C19	0.0491 (12)	0.0409 (11)	0.0411 (11)	0.0014 (9)	0.0133 (9)	0.0017 (9)
C20	0.0585 (14)	0.0431 (12)	0.0576 (13)	0.0089 (11)	0.0205 (11)	0.0024 (10)
C21	0.0632 (15)	0.0449 (12)	0.0590 (14)	0.0029 (11)	0.0293 (12)	−0.0069 (10)
C22	0.0617 (14)	0.0504 (13)	0.0410 (11)	−0.0039 (11)	0.0220 (10)	−0.0091 (9)
C23	0.0449 (12)	0.0409 (11)	0.0346 (10)	−0.0028 (9)	0.0113 (8)	−0.0029 (8)
C24	0.129 (3)	0.093 (2)	0.0289 (11)	0.0286 (19)	0.0165 (14)	0.0052 (12)
C25	0.0578 (13)	0.0454 (12)	0.0332 (10)	0.0175 (10)	0.0115 (9)	0.0047 (9)
C26	0.0612 (14)	0.0579 (14)	0.0376 (11)	0.0083 (11)	0.0131 (10)	0.0123 (10)
C27	0.0765 (18)	0.0567 (14)	0.0510 (13)	−0.0060 (13)	0.0083 (12)	0.0081 (11)
C28	0.0904 (19)	0.0514 (14)	0.0396 (12)	−0.0066 (13)	0.0034 (12)	0.0000 (10)
C29	0.0822 (17)	0.0513 (13)	0.0317 (10)	0.0077 (12)	0.0130 (11)	0.0030 (9)
C30	0.0558 (13)	0.0423 (11)	0.0353 (10)	0.0087 (10)	0.0113 (9)	0.0040 (8)
N1	0.0464 (10)	0.0380 (9)	0.0298 (8)	0.0029 (7)	0.0095 (7)	−0.0004 (6)
N2	0.0437 (9)	0.0429 (9)	0.0269 (7)	0.0040 (7)	0.0080 (7)	−0.0017 (7)
N3	0.0733 (15)	0.0994 (18)	0.0508 (12)	−0.0016 (13)	0.0207 (11)	0.0201 (12)
N4	0.161 (3)	0.0768 (17)	0.0483 (13)	−0.0366 (18)	−0.0017 (15)	−0.0009 (12)
N5	0.0672 (13)	0.0601 (12)	0.0426 (10)	0.0042 (10)	0.0139 (9)	0.0065 (9)
O1	0.0793 (11)	0.0561 (9)	0.0316 (7)	0.0080 (8)	0.0072 (7)	0.0024 (7)
O2	0.130 (2)	0.162 (2)	0.0567 (12)	−0.0289 (17)	0.0489 (13)	−0.0078 (13)
O3	0.173 (3)	0.117 (2)	0.1001 (18)	−0.0324 (18)	0.0585 (17)	0.0345 (15)
O4	0.143 (4)	0.078 (3)	0.078 (3)	−0.036 (3)	−0.020 (3)	0.005 (3)
O5	0.199 (8)	0.110 (5)	0.0367 (16)	−0.044 (5)	0.011 (3)	−0.0088 (19)
O6	0.114 (7)	0.118 (5)	0.066 (3)	−0.033 (5)	0.051 (4)	−0.009 (3)
O7	0.086 (4)	0.061 (2)	0.0554 (19)	−0.008 (2)	0.0120 (19)	−0.0028 (15)
O4A	0.21 (3)	0.086 (9)	0.071 (8)	−0.071 (14)	−0.008 (12)	−0.001 (7)
O5A	0.119 (13)	0.086 (7)	0.047 (4)	−0.017 (6)	0.007 (5)	0.005 (4)
O6A	0.071 (8)	0.080 (7)	0.046 (3)	−0.012 (5)	0.015 (3)	0.020 (3)
O7A	0.105 (12)	0.124 (14)	0.048 (4)	−0.051 (11)	0.005 (5)	0.002 (5)
O8	0.0731 (11)	0.0602 (9)	0.0280 (7)	0.0123 (8)	0.0106 (7)	−0.0008 (6)

### 2-(2-Methoxyphenyl)-4,5-bis(4-methylphenyl)-1H-imidazol-3-ium 2,4,6-trinitrophenolate Geometric parameters (Å, º)

**Table:**

C1—C9	1.362 (3)	C19—C20	1.378 (3)
C1—N2	1.380 (2)	C19—H19	0.9300
C1—C2	1.459 (3)	C20—C21	1.372 (3)
C2—C3	1.380 (3)	C20—H20	0.9300
C2—C8	1.381 (3)	C21—C22	1.374 (3)
C3—C4	1.377 (3)	C21—H21	0.9300
C3—H3	0.9300	C22—C23	1.387 (3)
C4—C5	1.369 (3)	C22—H22	0.9300
C4—H4	0.9300	C23—O8	1.362 (2)
C5—C7	1.367 (3)	C24—O8	1.423 (2)
C5—C6	1.498 (3)	C24—H24A	0.9600
C6—H6A	0.9600	C24—H24B	0.9600
C6—H6B	0.9600	C24—H24C	0.9600
C6—H6C	0.9600	C25—O1	1.246 (2)
C7—C8	1.375 (3)	C25—C26	1.441 (3)
C7—H7	0.9300	C25—C30	1.450 (3)
C8—H8	0.9300	C26—C27	1.361 (3)
C9—N1	1.384 (2)	C26—N3	1.458 (3)
C9—C10	1.473 (2)	C27—C28	1.379 (3)
C10—C11	1.372 (3)	C27—H27	0.9300
C10—C16	1.382 (3)	C28—C29	1.370 (3)
C11—C12	1.384 (3)	C28—N4	1.438 (3)
C11—H11	0.9300	C29—C30	1.359 (3)
C12—C13	1.365 (3)	C29—H29	0.9300
C12—H12	0.9300	C30—N5	1.459 (3)
C13—C15	1.368 (4)	N1—H1	0.8600
C13—C14	1.514 (3)	N2—H2	0.8600
C14—H14A	0.9600	N3—O2	1.207 (3)
C14—H14B	0.9600	N3—O3	1.211 (3)
C14—H14C	0.9600	N4—O4	1.238 (7)
C15—C16	1.378 (3)	N4—O4A	1.249 (12)
C15—H15	0.9300	N4—O5A	1.256 (9)
C16—H16	0.9300	N4—O5	1.268 (6)
C17—N2	1.329 (2)	N5—O7A	1.199 (9)
C17—N1	1.331 (2)	N5—O7	1.223 (5)
C17—C18	1.452 (3)	N5—O6	1.218 (5)
C18—C19	1.392 (3)	N5—O6A	1.231 (9)
C18—C23	1.402 (3)		
			
C9—C1—N2	105.23 (16)	C20—C19—H19	119.7
C9—C1—C2	133.45 (17)	C18—C19—H19	119.7
N2—C1—C2	121.32 (16)	C21—C20—C19	120.0 (2)
C3—C2—C8	116.81 (19)	C21—C20—H20	120.0
C3—C2—C1	121.19 (17)	C19—C20—H20	120.0
C8—C2—C1	122.00 (18)	C20—C21—C22	120.8 (2)
C4—C3—C2	120.98 (19)	C20—C21—H21	119.6
C4—C3—H3	119.5	C22—C21—H21	119.6
C2—C3—H3	119.5	C21—C22—C23	119.80 (19)
C5—C4—C3	122.3 (2)	C21—C22—H22	120.1
C5—C4—H4	118.8	C23—C22—H22	120.1
C3—C4—H4	118.8	O8—C23—C22	123.66 (17)
C7—C5—C4	116.5 (2)	O8—C23—C18	116.19 (16)
C7—C5—C6	121.3 (2)	C22—C23—C18	120.16 (19)
C4—C5—C6	122.2 (2)	O8—C24—H24A	109.5
C5—C6—H6A	109.5	O8—C24—H24B	109.5
C5—C6—H6B	109.5	H24A—C24—H24B	109.5
H6A—C6—H6B	109.5	O8—C24—H24C	109.5
C5—C6—H6C	109.5	H24A—C24—H24C	109.5
H6A—C6—H6C	109.5	H24B—C24—H24C	109.5
H6B—C6—H6C	109.5	O1—C25—C26	124.71 (19)
C5—C7—C8	122.3 (2)	O1—C25—C30	123.6 (2)
C5—C7—H7	118.9	C26—C25—C30	111.63 (18)
C8—C7—H7	118.9	C27—C26—C25	124.5 (2)
C7—C8—C2	121.1 (2)	C27—C26—N3	115.8 (2)
C7—C8—H8	119.4	C25—C26—N3	119.7 (2)
C2—C8—H8	119.4	C26—C27—C28	119.2 (2)
C1—C9—N1	106.68 (15)	C26—C27—H27	120.4
C1—C9—C10	134.34 (18)	C28—C27—H27	120.4
N1—C9—C10	118.94 (16)	C29—C28—C27	120.9 (2)
C11—C10—C16	118.21 (18)	C29—C28—N4	119.6 (2)
C11—C10—C9	120.59 (18)	C27—C28—N4	119.4 (2)
C16—C10—C9	121.02 (18)	C30—C29—C28	119.9 (2)
C10—C11—C12	120.3 (2)	C30—C29—H29	120.0
C10—C11—H11	119.8	C28—C29—H29	120.0
C12—C11—H11	119.8	C29—C30—C25	123.8 (2)
C13—C12—C11	121.6 (2)	C29—C30—N5	116.31 (19)
C13—C12—H12	119.2	C25—C30—N5	119.84 (18)
C11—C12—H12	119.2	C17—N1—C9	110.54 (16)
C12—C13—C15	117.9 (2)	C17—N1—H1	124.7
C12—C13—C14	120.7 (3)	C9—N1—H1	124.7
C15—C13—C14	121.4 (2)	C17—N2—C1	111.74 (15)
C13—C14—H14A	109.5	C17—N2—H2	124.1
C13—C14—H14B	109.5	C1—N2—H2	124.1
H14A—C14—H14B	109.5	O2—N3—O3	122.4 (2)
C13—C14—H14C	109.5	O2—N3—C26	119.1 (3)
H14A—C14—H14C	109.5	O3—N3—C26	118.4 (2)
H14B—C14—H14C	109.5	O4A—N4—O5A	123.9 (13)
C13—C15—C16	121.3 (2)	O4—N4—O5	124.7 (6)
C13—C15—H15	119.3	O4—N4—C28	119.3 (6)
C16—C15—H15	119.3	O4A—N4—C28	115.9 (13)
C15—C16—C10	120.6 (2)	O5A—N4—C28	118.5 (7)
C15—C16—H16	119.7	O5—N4—C28	116.0 (4)
C10—C16—H16	119.7	O7—N5—O6	122.4 (5)
N2—C17—N1	105.81 (16)	O7A—N5—O6A	122.8 (11)
N2—C17—C18	127.47 (16)	O7A—N5—C30	122.4 (8)
N1—C17—C18	126.70 (17)	O7—N5—C30	118.5 (4)
C19—C18—C23	118.59 (17)	O6—N5—C30	119.1 (4)
C19—C18—C17	120.09 (16)	O6A—N5—C30	114.8 (8)
C23—C18—C17	121.32 (17)	C23—O8—C24	119.02 (17)
C20—C19—C18	120.67 (19)		
			
C9—C1—C2—C3	−179.6 (2)	O1—C25—C26—C27	176.4 (2)
N2—C1—C2—C3	−0.1 (3)	C30—C25—C26—C27	−1.9 (3)
C9—C1—C2—C8	0.6 (4)	O1—C25—C26—N3	−4.5 (3)
N2—C1—C2—C8	−179.9 (2)	C30—C25—C26—N3	177.2 (2)
C8—C2—C3—C4	0.3 (3)	C25—C26—C27—C28	1.3 (4)
C1—C2—C3—C4	−179.5 (2)	N3—C26—C27—C28	−177.8 (2)
C2—C3—C4—C5	0.7 (3)	C26—C27—C28—C29	0.3 (4)
C3—C4—C5—C7	−1.2 (3)	C26—C27—C28—N4	178.3 (3)
C3—C4—C5—C6	−180.0 (2)	C27—C28—C29—C30	−1.1 (4)
C4—C5—C7—C8	0.7 (4)	N4—C28—C29—C30	−179.0 (2)
C6—C5—C7—C8	179.6 (3)	C28—C29—C30—C25	0.3 (4)
C5—C7—C8—C2	0.2 (5)	C28—C29—C30—N5	−179.6 (2)
C3—C2—C8—C7	−0.7 (4)	O1—C25—C30—C29	−177.2 (2)
C1—C2—C8—C7	179.1 (2)	C26—C25—C30—C29	1.1 (3)
N2—C1—C9—N1	−0.7 (2)	O1—C25—C30—N5	2.6 (3)
C2—C1—C9—N1	178.9 (2)	C26—C25—C30—N5	−179.02 (19)
N2—C1—C9—C10	176.9 (2)	N2—C17—N1—C9	−0.4 (2)
C2—C1—C9—C10	−3.5 (4)	C18—C17—N1—C9	177.88 (18)
C1—C9—C10—C11	−72.6 (3)	C1—C9—N1—C17	0.7 (2)
N1—C9—C10—C11	104.8 (2)	C10—C9—N1—C17	−177.36 (17)
C1—C9—C10—C16	112.4 (3)	N1—C17—N2—C1	−0.1 (2)
N1—C9—C10—C16	−70.3 (3)	C18—C17—N2—C1	−178.31 (18)
C16—C10—C11—C12	0.1 (4)	C9—C1—N2—C17	0.5 (2)
C9—C10—C11—C12	−175.1 (2)	C2—C1—N2—C17	−179.13 (17)
C10—C11—C12—C13	−0.2 (4)	C27—C26—N3—O2	153.3 (3)
C11—C12—C13—C15	0.2 (4)	C25—C26—N3—O2	−25.9 (4)
C11—C12—C13—C14	178.3 (3)	C27—C26—N3—O3	−26.5 (4)
C12—C13—C15—C16	−0.2 (4)	C25—C26—N3—O3	154.3 (3)
C14—C13—C15—C16	−178.3 (3)	C29—C28—N4—O4	168.8 (7)
C13—C15—C16—C10	0.2 (4)	C27—C28—N4—O4	−9.2 (8)
C11—C10—C16—C15	−0.1 (3)	C29—C28—N4—O4A	−163 (3)
C9—C10—C16—C15	175.1 (2)	C27—C28—N4—O4A	19 (3)
N2—C17—C18—C19	−177.60 (19)	C29—C28—N4—O5A	30.6 (17)
N1—C17—C18—C19	4.5 (3)	C27—C28—N4—O5A	−147.4 (17)
N2—C17—C18—C23	3.1 (3)	C29—C28—N4—O5	−14.3 (10)
N1—C17—C18—C23	−174.78 (18)	C27—C28—N4—O5	167.7 (10)
C23—C18—C19—C20	−0.1 (3)	C29—C30—N5—O7A	169 (3)
C17—C18—C19—C20	−179.47 (19)	C25—C30—N5—O7A	−11 (3)
C18—C19—C20—C21	0.1 (3)	C29—C30—N5—O7	−156.8 (6)
C19—C20—C21—C22	0.0 (3)	C25—C30—N5—O7	23.4 (7)
C20—C21—C22—C23	−0.1 (3)	C29—C30—N5—O6	21.8 (14)
C21—C22—C23—O8	−180.0 (2)	C25—C30—N5—O6	−158.1 (13)
C21—C22—C23—C18	0.1 (3)	C29—C30—N5—O6A	−10.5 (14)
C19—C18—C23—O8	−179.89 (17)	C25—C30—N5—O6A	169.7 (14)
C17—C18—C23—O8	−0.6 (3)	C22—C23—O8—C24	2.7 (3)
C19—C18—C23—C22	0.0 (3)	C18—C23—O8—C24	−177.4 (2)
C17—C18—C23—C22	179.34 (18)		

### 2-(2-Methoxyphenyl)-4,5-bis(4-methylphenyl)-1H-imidazol-3-ium 2,4,6-trinitrophenolate Hydrogen-bond geometry (Å, º)

**Table:**

*D*—H···*A*	*D*—H	H···*A*	*D*···*A*	*D*—H···*A*
N1—H1···O1	0.86	1.82	2.661 (2)	167
N2—H2···O8	0.86	2.05	2.601 (2)	122
